# Case report of severe coronary artery tortuosity with coexisting connective tissue disease

**DOI:** 10.1111/anec.13052

**Published:** 2023-02-10

**Authors:** Zao‐Xian Xu, Yi Yang, Shang‐Ping Xin, Rong‐Fang Zhou, Xiao‐Ling Shou

**Affiliations:** ^1^ Department of Cardiac Rehabilitation Zhe Jiang Hospital Zhejiang China

**Keywords:** connective tissue disease, coronary artery tortuosity, electron‐beam computed tomography angiography

## Abstract

Coronary artery tortuosity (CAT) is frequently detected during coronary angiography or coronary electron‐beam computed tomography angiography by cardiovascular interventionalists. In this article, we described the case of a 69‐year‐old female patient with recurrent chest discomfort for 1 month and recurrence 1 week ago, accompanied by emaciation, gastrointestinal discomfort, and low skin temperature at the extremities. After a series of tests, the patient was finally diagnosed with severe CAT and coexisting connective tissue disease. Accordingly, she was treated with conventional medications, and diet and lifestyle modifications. The symptoms of the patient resolved gradually after 1 year of follow‐up. Although there is no unanimous conclusion on the pathogenesis and clinical characteristics of CAT, this disease may provide a clue to the diagnosis of connective tissue disease, and warrants exploration through further research.

## Graphical abstract

The patient was hospitalized due to “chest distress,” and a severe tortuous coronary artery was found after coronary CTA, in which the left anterior descending branch and circumvolute branch were the main ones. After reviewing a large amount of literature, the common etiology, imaging manifestations, diagnostic criteria and Therapeutic schedule of coronary tortuous arteries were further summarized. At the same time, combined with the patient's clinical manifestations, examination and past history, the patient was diagnosed as connective tissue disease after multidisciplinary discussion.

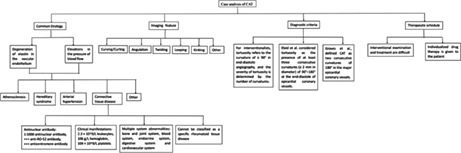

## 1 INTRODUCTION

Cardiovascular interventionalists often encounter coronary artery tortuosity (CAT) while performing coronary angiography or coronary computed tomography (CT) angiography, which, in most reports, is considered as resulting from changes in the vessel wall or blood flow within the lumen (Zegers et al., 2007). The clinical manifestations of CAT are varied—they commonly include curving/curling, angulation, twisting, looping, and kinking of vessels (Han, 2012), and there is no clear consensus on the manifestations. In this article, we report a case of severe CAT with coexisting connective tissue disease. It may be noted that in some literature from China and abroad, CAT is considered a manifestation of connective tissue disease in some cases.

## 2 CASE DETAILS

**FIGURE 1:**
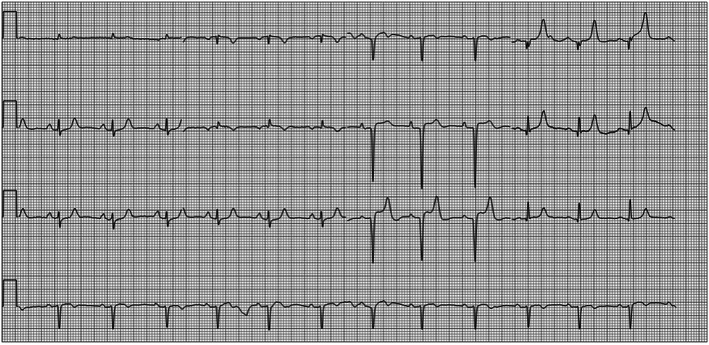
Electrocardiogram. Conclusion: (1) Sinus rhythm; (2) Abnormal initial segment of V_2_–V_5_ QRS.

**FIGURE 2:**
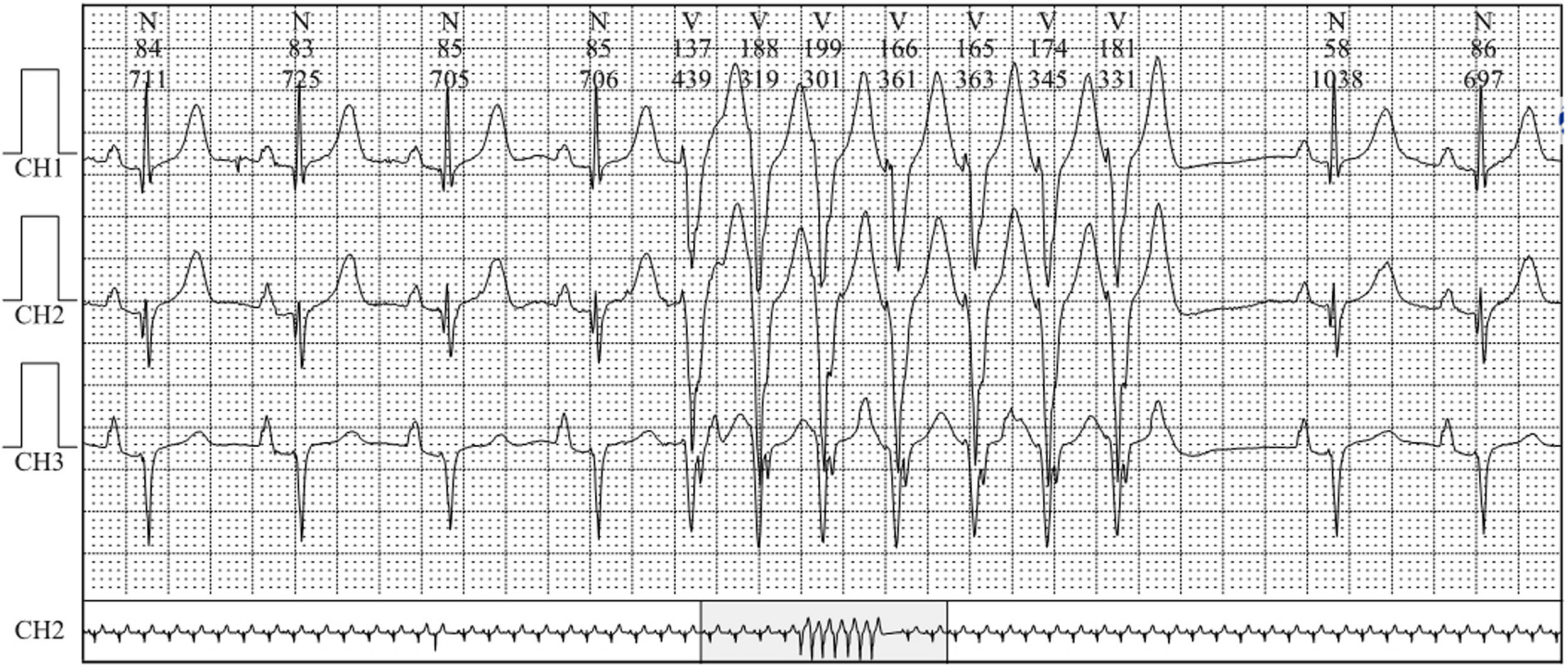
Dynamic electrocardiogram. Conclusion: Short runs of ventricular tachycardia with seven consecutive episodes at 178 beats/min.

**FIGURE 3:**
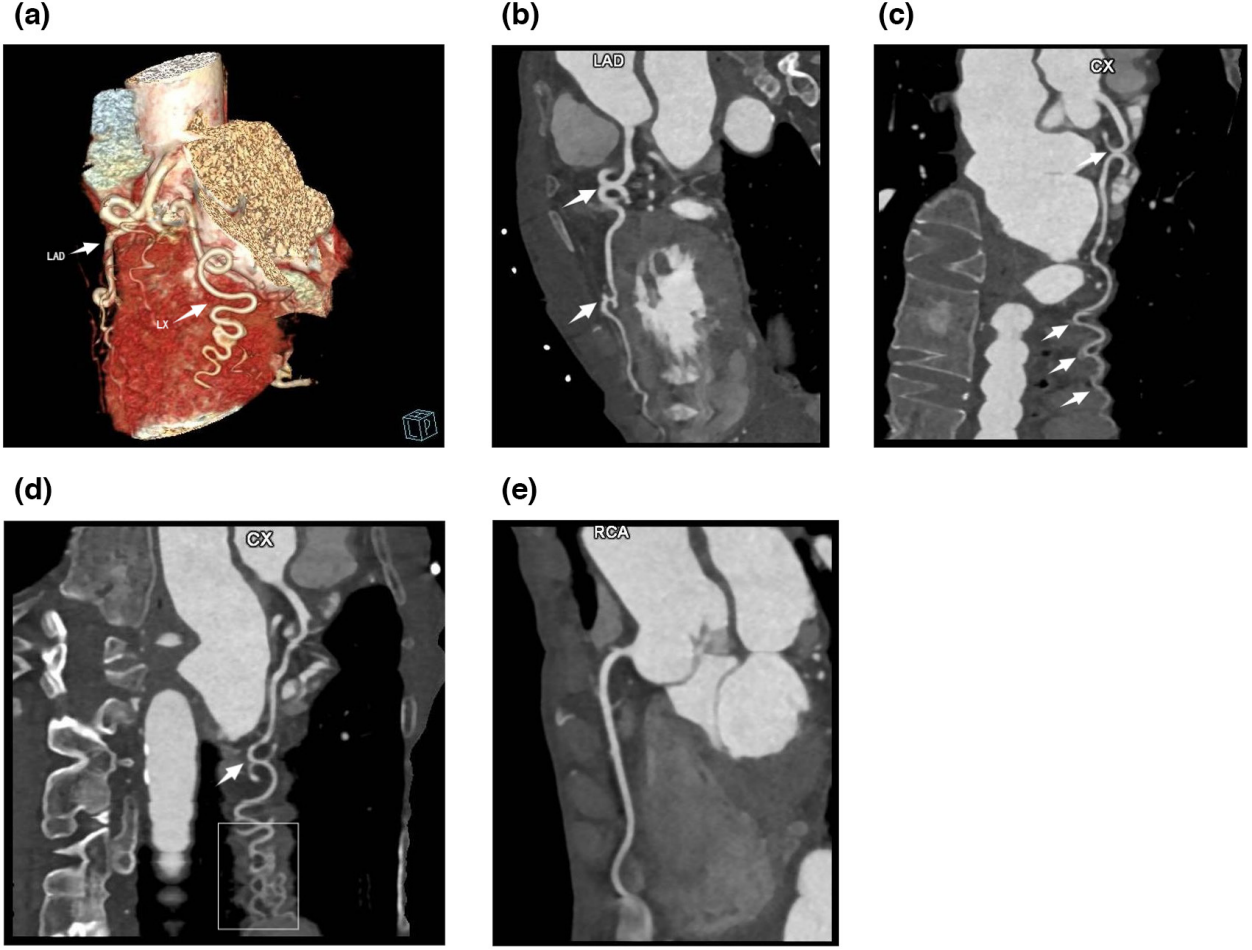
(a–e) Coronary computed tomography angiogram. Results: Severe coronary artery tortuosity with the most pronounced presentation in the circumflex branch, a localized non‐calcified plaque at the middle of the left anterior descending branch, and slight stenosis of the corresponding lumen.

A 69‐year‐old woman visited the outpatient department of our hospital due to recurrent chest discomfort for 1 month and recurrence 1 week ago. The patient recalled persistent chest tightness and occasional chest pain in the precordial region, which lasted for approximately 5 min and then relieved spontaneously. This symptom was not severe and could be tolerated and was relieved when resting and was usually related to the weather, without other manifestations such as dizziness, palpitation, and dyspnea. The patient reported a history of sleep disorder without intervention with sleeping pills, recurrent conjunctival hemorrhage that was intermittently treated with medication, chronic non‐atrophic gastritis with erosion for more than 30 years that was intermittently treated with Traditional Chinese Medicine and Western medicine, hysterectomy 20 years ago, tuberculosis 30 years ago that had been cured, trauma involving mutilation of the right index and middle fingers, and penicillin allergy that manifested as chest tightness. The results of the physical examination were—height of 159 cm, weight of 35 kg, a calculated body mass index of 13.8 kg/m^2^, emaciation, blood pressure, and heart rate within normal limits, joint deformities in both hands without joint swelling or stiffness, and low skin temperature. Laboratory tests showed normal myocardial enzymes and troponin I, 1.84 mmol/L low‐density lipoprotein cholesterol, 2.7 mmol/L high‐density lipoprotein cholesterol, 2.3 × 10^9^/L leukocytes, 106 g/L hemoglobin, 104 × 10^9^/L platelets, 1:1000 antinuclear antibody, +++ anti‐RO‐52 antibody, +++ anticentromere antibody, normal anti‐cyclic citrullinated peptide antibody, 32 mm/h erythrocyte sedimentation rate, and 15 mg/L C‐reactive protein. The thyroid function test showed 7.174 μIU/mL thyroid‐stimulating hormone. The electrocardiogram (ECG) indicated sinus rhythm with the abnormal initial segment of V_2_–V_5_ QRS (Figure 1). The echocardiogram was normal. The treadmill exercise test was terminated early because of poor exercise endurance, and no significant changes were observed in the ST‐T segment. The dynamic ECG revealed short runs of ventricular tachycardia with seven consecutive episodes at 178 beats/min (Figure 2). In addition, ambulatory blood pressure was normal. Chest CT showed multiple old lesions in both lungs, left upper pleural hypertrophy, localized calcification of the aorta, and posterior right tracheal diverticulum. The pulmonary function test suggested moderate restrictive pulmonary ventilation dysfunction, and the result of the bronchial dilation test was negative. Coronary CT angiography findings showed severe CAT with the most pronounced presentation in the circumflex branch, a localized non‐calcified plaque at the middle of the left anterior descending branch, and slight stenosis of the corresponding lumen (Figure 3a–e). Additionally, the patient was diagnosed with connective tissue disease.

## 3 DISCUSSION

Coronary artery tortuosity is often detected during coronary angiography or coronary electron‐beam computed tomography angiography by cardiovascular interventionalists. The pathogenesis and clinical characteristics of CAT have been extensively reported. Nevertheless, no consensus has been reached so far. In most reports, the etiology of CAT is shown to be associated with changes in mechanical forces including traction, pressure, and retraction forces within the lumen caused by alterations in the vessel wall or blood flow within the lumen. It must be noted that these changes in mechanical forces cause a gradual decrease in the energy during blood flow within the lumen, that is, the conversion of potential energy to thermal energy, to reduce the rate of distal blood flow, thus contributing to distal myocardial ischemia (Zegers et al., 2007). Clinically, CAT is commonly correlated with etiology such as age, atherosclerosis, hereditary syndrome, and arterial hypertension (Han, 2012). The main mechanisms of CAT include degeneration of elastin in the vascular endothelium and elevations in the pressure of blood flow in the lumen (Zegers et al., 2007).

Coronary artery tortuosity has diverse clinical manifestations, commonly including curving/curling, angulation, twisting, looping, and kinking of vessels (Han, 2012). Currently, the diagnostic criteria for “tortuosity” are not yet clearly established, and many scholars have put forward their opinions in this regard. For interventionalists, tortuosity refers to the curvature of ≥90° in end‐diastolic angiography, and the severity of tortuosity is determined by the number of curvatures (Alderman & Stadius, 1992). In addition, Eleid et al. (2014) considered tortuosity as the presence of at least three consecutive curvatures (≥2 mm in diameter) of 90°–180° at the end‐diastole of epicardial coronary vessels. Groves et al. (2009) defined CAT as two consecutive curvatures of 180° in the major epicardial coronary vessels. Li et al. (2011) concluded that tortuosity was defined as the presence of three curvatures (≥45° change in vessel orientation) in at least one major vessel during systole and diastole.

Coronary artery tortuosity has been proposed by some scholars as one of the pathophysiological mechanisms of myocardial ischemia in patients without coronary stenosis, although it has no uniform diagnostic criteria in China and abroad so far (Estrada et al., 2017; Mihai et al., 2021). Li et al. (2012) observed that the incidence of myocardial perfusion defects was higher in the CAT group than in the non‐CAT group. Since CAT complicates interventional procedures, the pressure changes of blood flow in the tortuous vessels cannot be effectively measured by invasive catheters, and conventional radionuclide myocardial perfusion imaging cannot exclude coronary microvascular disease. Hence, the extent of the impact of CAT on myocardial ischemia is unclear (Parekh et al., 2014). During percutaneous coronary angiography, tortuous vessels interfere with the performance of intracoronary ultrasound, optical coherence tomography, directional and rotational atherectomy, and the assessment of coronary artery pressure and measurement of microvascular resistance index with fractional flow reserve, thus resulting in increased difficulty of the surgery, excessive intraoperative contrast and radiation, elevated surgery risk, and decreased surgery success rates (Ellis et al., 1990; Parekh et al., 2014). The failure rate of surgery is 15%–40% depending on the severity of the tortuosity (Ellis et al., 1990).

In the present case, the patient had a final diagnosis of severe CAT with connective tissue disease. Based on electrocardiography and dynamic electrocardiography along with radionuclide myocardial perfusion imaging, we considered the presence of myocardial ischemia. However, the patient refused further coronary angiography and radionuclide myocardial perfusion imaging. With advice from experts in the Rheumatology and Immunology Department and the Nutrition Department, the patient was given antiplatelet drugs and β‐receptor blockers, with advice on modifications of daily diet and lifestyle. After 1 year of follow‐up, the patient reported significant relief with respect to the chest tightness.

Recently, vascular diseases are also considered a common type of lesion in connective tissue disease, which can manifest as inflammation and thickening of the vessel wall and stenosis of the lumen. It has been reported in China and internationally that in certain cases, CAT may be considered a manifestation of connective tissue disease or also serve as a diagnostic clue for coexisting vascular diseases such as fibromuscular dysplasia or spontaneous coronary artery dissection (Kahe et al., 2020). Among vascular diseases, artery tortuosity syndrome is a rare autosomal recessive connective tissue disease, which is majorly characterized by elongation and tortuosity of the middle/large arteries (Ajayan et al., 2022). In addition, it has also been reported that recurrent infections and connective tissue abnormalities lead to tortuosity, dilation, and aneurysm in coronary vessels in patients with hyper‐Immunoglobulin E (IgE) syndrome induced by autosomal dominant STAT3 mutations, with tortuosity and dilation occurring in 70% of the 38 patients with hyper‐IgE syndrome (Freeman et al., 2011). Loeys‐Dietz syndrome is a connective tissue disease characterized by aortic aneurysms, artery tortuosity, and aortic dissection, which predisposes to spontaneous coronary artery dissection (Lin et al., 2022; Solomonica et al., 2019).

The clinical treatment and prognosis of CAT remain to be explored, and the pharmacological or surgical interventions need to be determined based on the condition. Nevertheless, most experts and scholars currently believe that tortuosity is a benign lesion that does not require specific treatment or intervention. Therefore, more studies are warranted to further probe the pathogenesis and clinical features of CAT to address the differing opinions surrounding the treatment of this condition.

## 4 AUTHOR CONTRIBUTIONS

Conception and design of the research: Zao‐Xian Xu. Acquisition of date: Yi Yang, Shang‐Ping Xin. Analysis and interpretation of the date: Zao‐Xian Xu, Xiao‐Ling Shou, Yi Yang, Shang‐Ping Xin. Statistical analysis: No. Obtaining financing: Rong‐Fang Zhou. Writing of the manuscript: Zao‐Xian Xu. Critical revision of the manuscript for intellectual content: Zao‐Xian Xu, Xiao‐Ling Shou.

## FUNDING INFORMATION

Zhejiang Medical and Health Science and Technology Program “Study on Improvement of Myocardial Ischemia with Coronary Heart Disease by FFRCT Quantitative Exercise Rehabilitation” (2020KY389).

## CONFLICT OF INTEREST STATEMENT

The authors declare that they have no competing interests.

## ETHICS APPROVAL

The study was conducted in accordance with the Declaration of Helsinki (as was revised in 2013). The study was approved by the Ethics Committee of the Zhe Jiang Hospital (approval no. 2020 Provisional Trial no. 55K).

## CONSENT TO PARTICIPATE

Written informed consent was obtained from all participants.

## Data Availability

The data that support the findings of this study are available from the corresponding author upon reasonable request.
